# Double Lumen Endobronchial Tube Placement for Lung Separation via a Tracheostomy Stoma in a Patient Status-Post Laryngectomy

**DOI:** 10.7759/cureus.39858

**Published:** 2023-06-02

**Authors:** Alex Wolfram, Joseph Whitmore, Daniel Haines, Ryan Grell

**Affiliations:** 1 Anesthesiology and Perioperative Medicine, University of Louisville School of Medicine, Louisville, USA

**Keywords:** tracheostomy, : surgical tracheostomy, double lumen tube, difficult airway management, tracheal stoma, double-lumen endobronchial tube, lung cancer surgery

## Abstract

A 63-year-old male with a history of chronic obstructive pulmonary disease and squamous cell carcinoma of the larynx status-post laryngectomy and tracheostomy presented for a robotic-assisted right upper lobectomy for neoplasm excision. On physical examination, he was noted to have moderate hypoxia with an SpO_2 _of 93% on room air. In order to facilitate potential apneic oxygen insufflation and continuous positive airway pressure in the operative lung, a traditional left-sided 35-French double-lumen endobronchial tube was placed through his tracheostomy, and utilized to facilitate lung separation and to improve surgical manipulation. The patient tolerated the procedure well and was extubated to a tracheostomy collar with a 100% fraction of inspired oxygen delivered with 15 liters per minute of flow.

## Introduction

The placement of oral double-lumen endobronchial tubes (DLT) is a routine task for a general anesthesiologist; however, the absence of a larynx makes orotracheal intubation impossible [1,2]. Although specialized DLTs, which feature substantial length and angular differences, exist for utilization in patients with tracheostomies, they are not approved for use in the United States. The primary method of lung separation and isolation situations tends to be a bronchial blocker; however, many of these devices do not allow for the provision of apneic oxygen insufflation and continuous positive airway pressure in the operative lung [3]. Here, we present a case where those options needed to be available due to baseline hypoxia in our patient.

## Case presentation

A 63-year-old male with a history of chronic obstructive pulmonary disease (COPD) and squamous cell carcinoma of the larynx status-post laryngectomy and tracheostomy presented for a robotic-assisted thoracoscopic right upper lobectomy for neoplasm excision. Approximately three months prior to his presentation for surgery, he was found to have a suspicious mass incidentally noted on a computed tomography scan that was performed after a motor vehicle accident. Appropriate imaging scans were performed for cancer staging, and the mass was subsequently biopsied using moderate sedation. The tumor was found to be positive for stage 1 adenocarcinoma, and the patient was scheduled for surgery.

On physical examination on the day of surgery, he was alert, oriented, and in no acute distress. He was noted to be in sinus rhythm with a blood pressure of 141/92, heart rate of 74 beats per minute, respiratory rate of 12 breaths per minute via a 7.0 mm single lumen cuffed tracheostomy tube, oxygen saturation of 93% on room air, and a temperature of 98.8°F. The patient’s height was 175 centimeters and weight was 54 kilograms. He was able to achieve approximately three metabolic equivalents.

His preoperative laboratory results were notable for a hemoglobin of 10.8 g/dL (normal reference range 13-17.5 g/dL), platelet count of 187 x 10^3^ mm^-3^, and an international normalized ratio (INR) of 1.18. A type and cross was obtained, and two units of packed red blood cells were reserved. A transthoracic echocardiogram from three months prior was notable for a left ventricular ejection fraction of 42% with mild global hypokinesis. A subsequent left heart catheterization was notable for nonobstructive coronary artery disease in multiple vessels.

In the preoperative area, a right radial arterial line and two large bore peripheral intravenous catheters were placed using 1% lidocaine injections for analgesia. The patient was then transported to the operating room and connected to the American Society of Anesthesiologists standard monitors. The patient was preoxygenated with 100% fraction of inspired oxygen (FiO_2_) for greater than two minutes via the tracheostomy tube connected to the anesthesia machine. Once the expiratory concentration of oxygen (EtO_2_) was measured to be greater than 90%, general anesthesia was induced using 120 mg of propofol, 100 mcg of fentanyl, and 50 mg of rocuronium. The trachea and mainstem bronchi were then quickly inspected using a pediatric fiberoptic bronchoscope to rule out unanticipated obstruction and to aid in the intubation planning process. 

**Figure 1:**
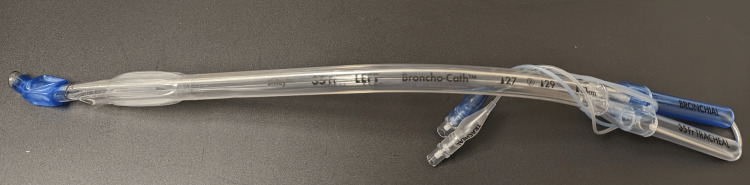
Traditional DLT

**Figure 2:**
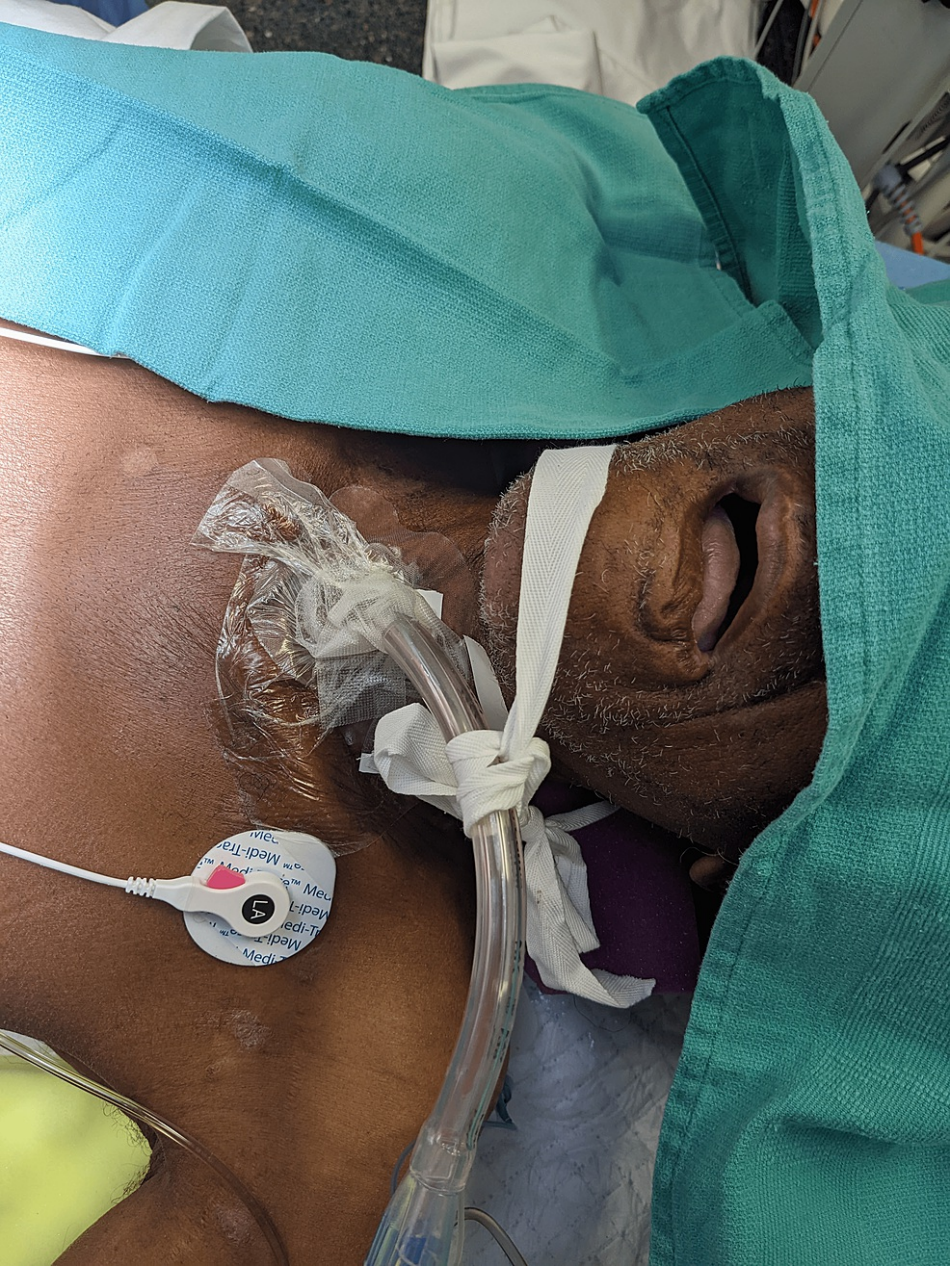
Image showing DLT placement via tracheostomy stoma

After sufficiently manually ventilating the patient until he regained EtO_2_ of greater than 90%, his tracheostomy tube was removed, and he was then intubated through his tracheostomy stoma using a left-sided 35-French double lumen endobronchial tube (DLT) (Figure 1). A pediatric fiberoptic bronchoscope was utilized as a stylet and as a means for confirmation of proper placement. The DLT was connected to the anesthesia machine and the presence of end-tidal carbon dioxide and adequate tidal volumes were confirmed. The DLT was then secured with cloth tracheostomy ties and tape (Figure 2). Proper tube position was again confirmed after positioning in the left lateral decubitus position. Lung separation was achieved by occluding the tracheal lumen of the DLT which provided an adequate operative field for the surgeons. 

The surgery was quickly and uneventfully completed without any surgical or anesthetic complications. The patient tolerated one lung ventilation well requiring an FiO_2_ of less than 50% and a positive end-expiratory pressure of 5 cm H_2_0 on the nonoperative lung for the entirety of the case. Intraoperative arterial blood gases remained stable with no significant findings. At the conclusion of the case, the patient was moved to the supine position, his neuromuscular blockade was reversed using sugammadex, and his DLT was exchanged for a new tracheostomy tube. Recruitment maneuvers were performed, and a chest x-ray was taken to ensure operative lung reinflation. After ensuring adequate minute ventilation, he was placed on a tracheostomy collar with 100% FiO_2_ delivered with 15 liters per minute of flow, and transported to the post-anesthesia care unit where he was observed for approximately 90 minutes before being transferred to the hospital ward in stable condition. He was discharged home in stable condition two days later.

## Discussion

Many procedures in the thoracic cavity require one lung ventilation (OLV) in order to improve surgical visualization, reduce the likelihood of contaminating a healthy lung with a diseased lung, or reduce the chance of rupturing a lung bleb. Lung isolation and separation can be achieved through three main mechanisms: placement of double-lumen endobronchial tubes (DLTs), bronchial blockers (BB), or main-stemmed single-lumen endotracheal tubes (MSLT) [1,2]. Each of these techniques has significant advantages and disadvantages. Patient comorbidities, anticipated difficulty with placement, anticipated need for intervention on the operative lung, and surgeon/anesthesiologist preference often influence this decision.

DLTs are most commonly used because of their ease of placement, rare requirement of repositioning, and ability to provide continuous positive airway pressure (CPAP) or suction to the operative lung [1-3]. Unfortunately, by their nature, DLTs are significantly larger than their single-lumen endotracheal tube (SLT) relatives. As a result, they are more likely to cause sore throat, hoarseness, and airway injuries. Due to increased flow resistance, size, and noxious stimulation, DLTs are not routinely utilized for post-procedure ventilation [4]. This often necessitates an exchange from a DLT to a SLT in patients who require post-operative ventilation which can be challenging after a prolonged surgery or in patients with difficult airways [5]. 

Placing a BB is the second most common method of obtaining lung separation and isolation [6]. Advantages of a BB include the ability to be placed via an existing SLT or tracheostomy, isolate a specific lobe, avoid the requirement of exchanging for an SLT at the end of the procedure, and minimize airway trauma [3,6]. Disadvantages of BBs include a longer time to place, more frequently-required repositioning, and the inability to provide CPAP, apneic oxygen insufflation, suctioning, and bronchoscopy to the operative lung.

MSLTs are also infrequently utilized for achieving OLV. MSLTs are simply SLTs that have been advanced into one of the mainstem bronchi. The advantages of this method are quite sparse. MSLTs are typically quite fast to place in the right lung due to the more favorable angle of the right mainstem bronchus takeoff compared to the left mainstem takeoff [7]. Frequently, existing SLTs can be sufficiently advanced to become MSLTs. With an SLT, airway trauma is also minimized. Unfortunately, MSLTs suffer from similar disadvantages as BBs. It is not possible to provide CPAP, apneic oxygen insufflation, suctioning, or bronchoscopy to the operative lung. It also can be quite challenging to place an MSLT in the left main bronchus and to avoid occluding the takeoff of the right upper lobe if it is placed on the right side. As a result, the utility of MSLTs is limited.

Patients who have previously undergone a laryngectomy and tracheostomy present a challenge for airway management during cases that require OLV. Exchanging a tracheostomy tube can cause bleeding or the creation of a false passage, especially in tracheostomies less than a week old. The altered anatomy leads to a higher rate of DLT and BB malposition, and subsequent difficulty maintaining adequate lung separation and isolation [1,2]. Additionally, standard DLTs can be too long for the shortened upper airway, too wide to fit through a stoma, or feature too narrow of an angle to easily position correctly. These challenges make BBs, placed via an existing cuffed tracheostomy or an SLT, more commonly used in these patients. However, as described above, BBs preclude oxygenation interventions on the operative lung [8,9].

**Figure 3:**
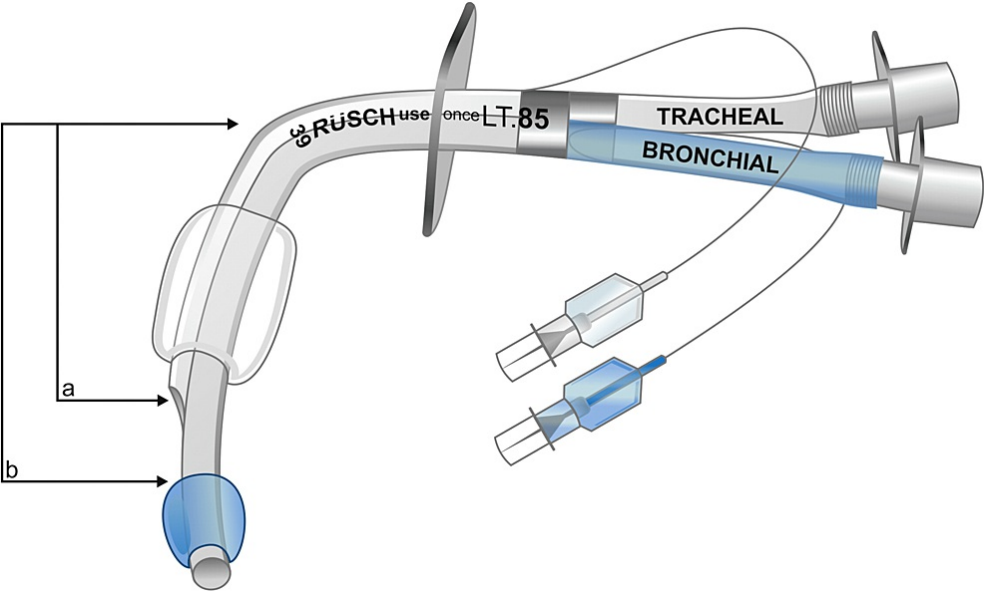
Rüsch Tracheopart® DLT (Teleflex Medical Europe Ltd., Athlone, Co, Westmeath, Ireland) "a" and "b" demonstrate sections of variable length based on DLT size [10] Reprinted from JCVA, 29(3), Dincq AS, Double-Lumen Tubes for Tracheostomized Patients, e35-e36, Copyright (2015), with permission from Elsevier.

Designed to solve this problem, specific DLTs for patients with tracheostomies have been created. These DLTs, such as Rüsch Tracheopart® DLT (Teleflex Medical Europe Ltd., Athlone, Co, Westmeath, Ireland) (Figure 3), are available in Europe. However, they are not yet approved for utilization in the United States [10]. The Tracheopart® is available in three lengths to accommodate patient-specific differences in the length between the carina and the tracheostomy. It also is available in a left- or right-sided configuration. The Tracheopart® differs from standard oral double-lumen endotracheal tubes in that it is shorter and has a significant curvature beginning at the insertion site of the trachea and ending at the distal end of the bronchial lumen. This typically results in a more secure fit with a lower incidence of tube migration with positional changes [10]. Unfortunately, even when available in a region, these devices tend to be more costly and more infrequently stocked in most operating rooms compared to traditional DLTs.

## Conclusions

Airway management in patients with laryngectomies can be quite complex. Appropriate device selection should be guided by the patient anatomy, equipment availability, surgeon and anesthesiologist preference, and the potential need for apneic oxygen insufflation, continuous positive airway pressure, and suctioning in the operative lung. In the absence of specific DLTs made for patients with tracheostomies, traditional DLTs can be considered for lung separation and isolation if the availability of these interventions is desired.
